# IL-17 inhibitor-associated inflammatory bowel disease: A study based on literature and database analysis

**DOI:** 10.3389/fphar.2023.1124628

**Published:** 2023-03-23

**Authors:** Zhenzhen Deng, Shengfeng Wang, Cuifang Wu, Chunjiang Wang

**Affiliations:** Department of Pharmacy, The Third Xiangya Hospital, Central South University, Changsha, Hunan, China

**Keywords:** IL-17 inhibitors, inflammatory bowel disease, FAERS database, pharmacovigilance, drug safety

## Abstract

**Objective:** Few real-world studies have shown clear association between interleukin (IL)-17 inhibitors and inflammatory bowel disease (IBD) onset. This study investigated the reporting prevalence and evaluated the clinical features and management of IL-17 inhibitor-related IBD events.

**Methods:** We used the US FDA Adverse Event Reporting System database and retrieved data, from 2015 to 2022, on IL-17 inhibitors to identify gastrointestinal inflammatory events and conduct disproportionality analyses by estimating the reporting odds ratios (RORs) and corresponding 95% confidence intervals (CIs). Furthermore, case reports and case series, from 2015 to 30 November 2022, on IBD induced by IL-17 inhibitors were collected for retrospective analysis.

**Results:** A total of 388 cases of primary suspected IL-17 inhibitor-associated gastrointestinal events were reported (268 IBD and 120 colitis), including 348 cases involving secukinumab (SEC), 36 cases involving ixekizumab (IXE), and 4 cases involving brodalumab (BRO). Statistically significant reporting rates of total IBD events were observed for SEC and IXE (ROR = 2.13, 95% CI [1.96-2.30] and ROR = 2.79, 95% CI [2.39-3.27], respectively), whereas BRO did not trigger a safety signal. Twenty-nine studies, which included 34 cases, showed evidence of IBD, following SEC (79.4%) and IXE (20.6%) treatment. The median age was 42 years; typical initial symptoms included diarrhea (90.9%), abdominal pain (57.6%), bloody diarrhea (51.5%), and fever (36.4%). The median time to onset of IBD symptoms was 2.9 months. Some cases were accompanied by elevated white blood cell (WBC) count (87.5%), erythrocyte sedimentation rate (ESR; 85.7%), C-reactive protein (CRP; 100%), and fecal calprotectin (FC; 100%). Cessation of IL-17 inhibitors plus treatment with corticosteroids and TNF antagonists, as either monotherapy or in combination, could lead to complete clinical remission. The median time to remission after IL-17 inhibitor discontinuation was 4 weeks.

**Conclusion:** IL-17 inhibitor treatment is associated with exacerbation and new onset of IBD and colitis. Obtaining a detailed patient history before initiation of treatment and monitoring gastrointestinal symptoms and intestinal inflammatory biomarkers during IL-17 inhibitor treatment is important for safe use of these drugs.

## Introduction

Interleukin (IL)-17 is a significant proinflammatory cytokine and a therapeutic target for rheumatological diseases as blocking IL-17 reduces disease activity and improves patient quality of life (Ouyang et al., 2008). The anti-IL-17 agents secukinumab (SEC), ixekizumab (IXE), and brodalumab (BRO), which were launched in January 2015, April 2016, and July 2017, respectively, are monoclonal IgG4 antibodies directed against IL-17A (SEC and IXE), or against the IL-17 receptor (BRO) (Langley et al., 2014; Griffiths et al., 2015; Lebwohl et al., 2015), and have been used to treat various autoimmune disorders, such as psoriasis (Pso), psoriatic arthritis (PsA), ankylosing spondylitis (AS), and axial spondyloarthritis (ax-Spa) (van der Heijde et al., 2017; Menter et al., 2019; Gossec et al., 2020; Wendling et al., 2022; Ramiro et al., 2023). Promising efficacy results in dermatology and rheumatology prompted the evaluation of these drugs in treatment of inflammatory bowel disease (IBD). Unfortunately, they were ineffective and induced paradoxical events (disease exacerbation after treatment with a theoretically curative drug) that prevented approval for treatment of patients with IBD. Increasing cases of induction or exacerbation of IBD have been reported among patients treated with drugs inhibiting IL-17 (Caron et al., 2022; Burisch et al., 2020), which suggests that rheumatologists and dermatologists should increase awareness of the risk and improve safety measures to prevent drug-induced IBD, as well as evaluate patients carefully to identify any contraindications before the initiation of IL-17 inhibitor therapy for psoriasis and/or rheumatological diseases (Felice et al., 2019).

To date, the pathophysiological mechanisms underlying these paradoxical effects are not well-defined, there is limited real-world data demonstrating the association between IL-17 inhibitor therapy and the onset of IBD, and there are no clear guidelines for the management of patients who experience these adverse events. We aimed to assess differences in safety signals using the FDA Adverse Event Reporting System (FAERS) database, and describe characteristics, management, and prognosis of IBD flare or new onset in patients after anti-IL-17 drug therapy in a real-life setting, which provides valuable information about emerging gastrointestinal safety issues related to IL-17 inhibitors.

## Methods

### Pharmacovigilance study

In this study, we conducted a retrospective, disproportionality, pharmacovigilance analysis. Data from 2015 Quarter 1 (Q1) to 2022 Q3 were retrieved from the publicly available FAERS database in FDA website to evaluate the risk of gastrointestinal inflammatory conditions with the use of different IL-7 inhibitor therapies.

We assessed the use of IL-17 inhibitors in a large-scale population. Study drugs were IL-17 inhibitors on the market: SEC, IXE, and BRO. To identify IL-17 inhibitor-related records, both brand names and generic names were used. Furthermore, IBD and colitis case reports in FAERS were coded using the preferred term (PT) according to the Medical Dictionary for Regulatory Activities Terminology (MedDRA). Drugs were assigned a role (primary suspect, secondary suspect, concomitant, and interacting) by the person reporting the adverse drug reaction (ADR). ADRs were categorized into two main groups: 1) “IBD,” including reported diagnoses of ulcerative colitis (UC), Crohn’s disease (CD), and undifferentiated IBD (uIBD); 2) “colitis,” including reported diagnoses of colitis (microscopic, ischemic, eosinophilic, and undifferentiated), proctitis, and enteritis. The effects of disproportionality analysis were evaluated using the established pharmacovigilance index reporting odds ratio (ROR), which were calculated as (a*d)/(b*c) (Supplementary Table S1). In our study, an event was defined as significant when the lower limit of the 95% CI of the ROR >1 and there were at least three cases to define a signal (van Puijenbroek et al., 2002).

### Descriptive study

A comprehensive search of multiple electronic databases, including PubMed, Embase, Wanfang, China National Knowledge Infrastructure (CNKI), and China Biology Medicine disc (CBMdisc), from January 2015 to September 2022, regarding IL-17 inhibitor-induced IBD was conducted, with no language restrictions. The search terms were “secukinumab or Cosentyx”, “Ixekizumab or Taltz,” “Brodalumab or Siliq,” “Ulcerative colitis,” “Crohn’s disease,” and “Inflammatory bowel disease”. Case reports and case series were included, and reviews, mechanistic studies, animal studies, and articles for which the full text was not available were excluded. Two reviewers searched the literature independently and examined the relevant studies for further assessment of inclusion/exclusion criteria and to identify clinical characteristics; collected data included the region of patient location, age, sex, Indication of IL-17 inhibitors, medical history, time to onset, clinical manifestations, laboratory tests, histopathological examinations, treatment, and prognosis of IL-17 inhibitor-induced IBD. The time to onset of target IBD was defined as the time from the start date of IL-17 inhibitor administration to the onset of the associated IBD.

## Results

### IL-17 inhibitor-associated IBD in the FAERS database

**FIGURE 1:**
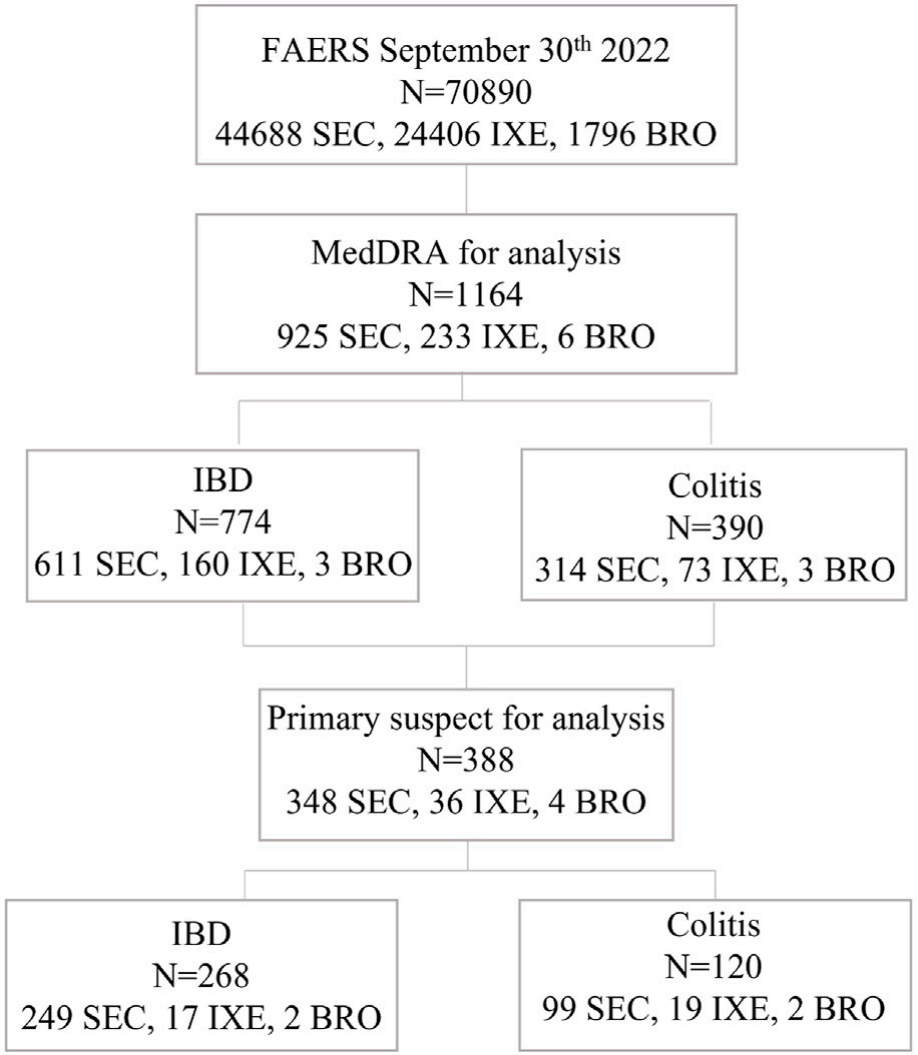
Flow chart of the study protocol. FAERS, FDA Adverse Event Reporting System; MedDRA, Medical Dictionary for Regulatory Activities Terminology; IBD, inflammatory bowel diseases; SEC, secukinumab, IXE, ixekizumab IXE; BRO, brodalumab.

Up to 2022 Q3, the numbers of reported adverse event (ADE) cases in the FAERS database related to SEC, IXE, and BRO were 44,688, 24,406, and 1796, respectively. From screening individual IL-17 inhibitor-related gastrointestinal inflammatory case reports, a total of 388 cases of primary suspect IL-17 inhibitor-associated inflammatory bowel disease and colitis were reported, of which 348 were SEC, 36 were IXE, and 4 were BRO. These cases included 268 IBD diagnoses and 120 colitis diagnoses (Figure 1). The main characteristics of the patients, disease seriousness, delay of onset, and disease outcomes are detailed in Table 1.

**TABLE 1:** Main characteristics of primary suspect cases in FAERS database.

**N**	**388**	**%**
Age (years)		
≤25	10	2.6
26–50	50	12.9
51–75	60	15.5
≥ 76	4	1.0
Unknown	264	68.0
Gender		
Female	171	44.1
Male	124	32.0
Unknown	93	23.9
Reporting country		
United Sates	222	57.2
Other countries	104	26.8
Unknown	62	16.0
Reporting year		
2015-2016	28	7.2
2017-2018	133	34.3
2019-2020	133	34.3
2021-2022	94	24.2
Anti-IL-17 as primary suspect drugs		
Secukinumab	348	89.7
Ixekizumab	36	9.3
Brodalumab	4	1.0
Outcome of IBD events		
Death	9	2.3
Life-threatening	6	1.5
Hospitalization-initial or prolonged	84	21.7
Disability	3	0.8
Required intervention	3	0.8
Other medical significant condition	224	57.7
Unknown	59	15.2
Reported indication for IL-17 inhibitors		
Ankylosing spondylitis	51	13.1
Psoriasis	100	25.8
Psoriatic arthropathy	53	13.7
Others	10	2.6
Unknown	174	44.8
Bowel disease		
All IBD	268	69.1
UC	87	32.5
CD	96	35.8
uIBD	85	31.7
All colitis	120	30.9
Onset time of symptoms, month		
<1	6	1.5
1-3	20	5.1
3–6	12	3.1
6–12	15	3.9
12–24	8	2.1
>24	9	2.3
Unknown	318	82.0

uIBD, Unclassified Inflammatory bowel disease; UC, ulcerative colitis; CD, Crohn’s disease; IL-17, Interleukin (IL)-17.

### Disproportionality analyses

From the entire database, the disproportionality analyses identified three unique preferred terms (PTs) for IBD and 12 PTs for colitis. Globally, all cases of IBD after SEC and IXE treatment accounted for 1.36% and 0.66% of all reported cases, respectively, and there was a statistically significant reporting rate of total IBD events observed among patients treated with SEC and IXE (ROR = 2.13, 95% CI [1.96–2.30] and ROR = 2.79, 95% CI [2.39-3.27], respectively), whereas this proportion was lower for BRO (0.17%; ROR = 1.48, 95% CI [0.48-4.6]) (Table 2). All colitis cases after SEC and IXE treatment accounted for 0.70% and 0.30% of all reported cases, respectively, and statistically significant reporting rates were also observed for total colitis events after SEC and IXE treatment (ROR = 1.60, 95% CI [1.43-1.79] and ROR = 1.96, 95% CI [1.56-2.47], respectively) (Table 3).

**TABLE 2:** Interleukin -17 inhibitors associated with Inflammatory bowel disease and the reporting odds ratios in FAERS database.

**PT**	**Secukinumab**		**Ixekizumab**		**Brodalumab**	
	**No. of AEs**	**ROR(95%CI** **^a^** **)**	**No. of AEs**	**ROR(95%CI** **^a^** **)**	**No. of AEs**	**ROR(95%CI** **^a^** **)**
Crohn’s disease	233	1.57^b^(1.38-1.79)	43	1.45^b^(1.08-1.96)	1	0.81 (0.11-5.73)
Colitis ulcerative	196	2.08^b^(1.81-2.40)	67	3.58^b^(2.81-4.55)	2	2.54 (0.63-10.17)
Inflammatory bowel disease	182	4.0^b^(3.43-4.61)	50	5.421^b^(4.10-7.17)	0	NA
Total events of interest	611	2.13^b^(1.96-2.30)	160	2.79^b^(2.39-3.27)	3	1.48 (0.48-4.6)

CI, confdence interval; NA, not available; ROR, reporting odds ratio; AEs, adverse events.

^a^ Two-sided CI for ROR.

^b^ ROR lower-bound CI values >1 and with ≥3 patients.

**TABLE 3:** Interleukin-17 inhibitors associated with Colitis and the reporting odds ratios in FAERS database.

**PT**	**Secukinumab**		**Ixekizumab**		**Brodalumab**	
	**No. of AEs**	**ROR(95%CI** **^a^** **)**	**No. of AEs**	**ROR(95%CI** **^a^** **)**	**No. of AEs**	**ROR(95%CI** **^a^** **)**
Colitis	223	2.09^b^(1.83-2.39)	53	2.50^b^(1.90-3.27)	3	3.37^b^(1.08-10.47)
Microscopic colitis	28	1.91^b^(1.32-2.78)	8	2.74^b^(1.37-5.49)	0	NA
Ischaemic colitis	10	0.41 (0.22-0.76)	2	0.41 (0.10-1.65)	0	NA
Proctitis	10	1.36 (0.73-2.54)	0	NA	0	NA
Proctitis ulcerative	4	1.93 (0.72-5.19)	0	NA	0	NA
Proctitis haemorrhagic	0	NA	1	18.92 (2.59-138.44)	0	NA
Enteritis	22	1.30 (0.85-1.97)	2	0.59 (0.15-2.37)	0	NA
Enterocolitis	10	0.61 (0.33-1.13)	1	0.31 (0.04-2.17)	0	NA
Enterocolitis haemorrhagic	5	0.82 (0.34-1.98)	4	3.34^b^(1.25-8.92)	0	NA
Eosinophilic colitis	1	0.70 (0.10-4.98)	1	3.54 (0.50-25.28)	0	NA
Colitis erosive	1	2.29 (0.32-16.59)	1	11.64 (1.61-84.21)	0	NA
Total events of interest	314	1.60^b^(1.43-1.79)	73	1.96^b^(1.56-2.47)	3	NA

CI, confdence interval; NA, not available; ROR, reporting odds ratio; AEs, adverse events.

^a^ Two-sided CI for ROR.

^b^ROR lower-bound CI values >1 and with ≥3 patients.

### Descriptive analysis

A total of 29 studies were identified in the published literature, including 25 case reports (Ehrlich et al., 2018; Grimaux et al., 2018; Paul et al., 2018; Wang et al., 2018; Achufusi et al., 2019; Grossberg, 2019; Haidari et al., 2019; Johnston and Veettil, 2019; Philipose et al., 2019; Rodriguez et al., 2019; Shimizu et al., 2019; Smith et al., 2019; Darch et al., 2020; Fieldhouse et al., 2020; Marin et al., 2020; Merino Gallego et al., 2020; Nazarian et al., 2020; Ali et al., 2021; Mu et al., 2021; Obeidat and Murakami, 2021; Ma et al., 2022; Sykaras et al., 2022; Kukol et al., 2019; Nallapeta et al., 2019; Sethi et al., 2019) and 4 case series (Fobelo et al., 2018; Shukla et al., 2018; Vernero et al., 2019; Lee et al., 2020); a total of 34 cases raised evidence of IBD occurrence during therapy with SEC (27 cases, 79.4%) and IXE (7 cases, 20.6%). Characteristics of the included cases are presented in Table 4. The patient sex ratio (male/female) was 1, the median age of included patients was 42 years (range 19–76 years), and 23 patients (67.7%) were between 26 and 50 years old. Data on race showed that 93.9% of patients (32 cases) were white, and two were Asian. Three patients (8.8%) had a prior history of IBD, and five patients (14.7%) had a family history of IBD. History of smoking was reported for eight patients (23.5%). The main indication for anti-IL-17 therapy was Pso (20 cases, 58.8%), followed by AS (7 cases, 20.6%), PsA (4 cases, 11.8%), and Pso with PsA (3 cases, 8.8%). Twenty-four patients had prior exposure to a TNF antagonist with inadequate response, of whom 13 received prior adalimumab and 11 received prior etanercept. The time between IL-17 inhibitor treatment start and symptom onset was assessable in 27 cases, showing a median time to onset of 2.9 months (range, 0.47–48 months); onset of symptoms occurred in 5 cases (18.5%) in less than a month, in 9 cases (33.3%) between 1 and 3 months, and in 10 cases (37.1%) between 3 and 12 months.

**TABLE 4:** Summary of demographic features of patients with interleukin (IL)-17 inhibitors-induced Inflammatory bowel disease.

**Variable**	**N**	**%**
Total	34	/
Data resource (*n* = 29)		
Case reports	25	86.2
Case series	4	13.8
Gender		
Female	17	50.0
Male	17	50.0
Age, years (*n* = 34)		
≤25	1	2.9
26–50	23	67.7
51–75	9	26.5
≥76	1	2.9
Reporter region (*n* = 33)		
USA	11	33.3
Europe	13	39.4
Canada	6	18.2
Asian	2	6.1
Other	1	3.0
IL-17 inhibitors (*n* = 34)		
Secukinumab	27	79.4
Ixekizumab	7	20.6
Pre-existing of IBD (*n* = 34)	3	8.8
New onset of IBD (*n* = 34)	31	91.2
Family history of IBD (*n* = 34)	5	14.7
Indication for IL-17 inhibitors (n = 34)		
PsO	20	58.8
PsA	4	11.8
Pso and PsA	3	8.8
AS	7	20.6
History of smoking (*n* = 34)		
Yes	8	23.5
No	4	11.8
Not mention	22	64.7
Prior exposure (with inadequate response) to TNF antagonist (*n* = 24)		
Adalimumab	13	54.2
Etanercept	11	45.8
Onset time of symptoms, months (*n* = 27)		
<1	5	18.5
1–3	9	33.3
3–6	7	25.9
6–12	3	11.1
12–24	2	7.4
>24	1	3.7

AS, ankylosing spondylitis; PsA, psoriatic arthritis; PsO, psoriasis; IBD, Inflammatory bowel disease; UC, ulcerative colitis; CD, Crohn’s disease.

### Clinical manifestations and laboratory and radiological features

Among the 34 cases included, IBD classification was reported in 28 cases, with 13 cases (46.4%) identified as UC, 10 cases (35.7%) as CD, and 5 cases (17.9%) as unclassified IBD. Symptoms were reported for 33 patients; the most common initial symptoms were diarrhea (30 cases, 90.9%), followed by abdominal pain (19 cases, 57.6%), bloody diarrhea (17 cases, 51.5%), and fever (12 cases, 36.4%). Other accompanying symptoms included weight loss (5 cases, 15.2%), chills (4 cases, 12.1%), and nausea (4 cases, 12.1%). One patient underwent colonoscopy for colorectal cancer screening, which revealed asymptomatic Crohn’s disease (Table 5).

**TABLE 5:** Clinical information of interleukin (IL)-17 inhibitors-induced Inflammatory bowel disease.

**Parameter**	**Clinical features**	**Value (%)**
IBD classify (*n* = 28)	UC	13 (46.4)
	CD	10 (35.7)
	Unclassified IBD	5 (17.9)
Initial symptoms (*n* = 33)	Diarrhea	30 (90.9)
	Bloody diarrhea	17 (51.5)
	Abdominal pain	19 (57.6)
	Fever	12 (36.4)
	Chills	4 (12.1)
	Nausea	4 (12.1)
	Weight loss	5 (15.2)
	Asymptomatic	1 (3.0)
WBC account (*10^9^/L, *n* = 8)	Normal	1 (12.5)
	Elevated	7 (87.5)
CRP (mg/L, *n* = 16)	Elevated	16 (100)
ESR (mm/hr, *n* = 7)	Normal	1 (14.3)
	Elevated	6 (85.7)
Fecal calprotectin (*n* = 4)	Elevated	4 (100)
Computed tomography examination (*n* = 10)	Normal	2 (20.0)
	Bowel wall thickening	5 (50.0)
	Pancolitis	2 (20.0)
	Colon perforated	1 (10.0)
Colonoscopy examination (*n* = 28)	Edematous	5 (17.9)
	Erythematous mucosa	6 (21.4)
	Hemorrhagic	3 (10.7)
	Friable mucosa	4 (14.3)
	Absent vascular pattern	4 (14.3)
	Superficial ulceration	6 (21.4)
	Deep ulcerations	13 (46.4)
	Active inflammation	8 (28.5)
Histopathology examination (*n* = 25)	Cryptitis	8 (32.0)
	Crypt abscesses	8 (32.0)
	Granulomatous lesions	7 (28.0)
	Chronic inflammation	11 (39.3)
Therapy (*n* = 31)	None	1 (3.2)
	Corticosteroids	10 (32.3)
	TNF antagonist	6 (19.4)
	Ustekinumab	2 (6.4)
	5-ASA	1 (3.2)
	Corticosteroids plus TNF antagonist	6 (19.4)
	Corticosteroids plus 5-ASA	4 (12.9)
	Corticosteroids plus Ustekinumab	1 (3.2)
Outcome of IBD event (*n* = 29)	Recovered/recovering	27(93.1)
	Relapse	2(6.9)
Time to symptom relief (*n* = 15)	Within 2 weeks	3 (20.0)
	2-4w	7 (46.7)
	>4w	5 (33.3)

IBD, Inflammatory bowel disease; UC, ulcerative colitis; CD, Crohn’s disease; WBC, white blood cell; ESR, erythrocyte sedimentation rate; CRP, C-reaction protein; TNF antagonist, Tumor Necrosis Factor antagonist; 5-ASA, 5-aminosalicylic acid.

The laboratory results for IL-17 inhibitor-induced IBD cases are summarized in Table 5. Elevated white blood cell count (WBC) was observed in seven patients (87.5%) out of eight reported cases, elevated erythrocyte sedimentation rate (ESR) levels were observed in six patients (85.7%), and increased C-reactive protein was reported in 16 patients (100%). Levels of fecal calprotectin were increased in four patients (100%).

Computed tomography (CT) results were reported for 10 patients. Typical imaging features were bowel wall thickening (5 cases, 50.0%), pancolitis (2 cases, 20.0%), and colon perforation (1 case, 10.0%); two patients showed normal CT results.

### Colonoscopy and histopathological examination

Colonoscopy results were reported for 28 patients, with deep ulcerations found in 13 patients (46.4%) and superficial ulcerations found in 6 patients (21.4%); eight patients (28.5%) showed active inflammation, four patients (14.3%) had friable mucosa, and four patients (14.3%) had loss of the normal vascular pattern. Intestinal mucosal biopsies were performed in 25 patients, and the characteristics of histopathological examination presented mainly as chronic inflammation (11 cases, 39.3%), cryptitis (8 cases, 32.0%), crypt abscesses (8 cases, 32.0%), and granulomatous lesions (7 cases, 28.0%).

### Treatment and prognosis

A total of 31 patients discontinued IL-17 inhibitor therapy after the onset of IBD. Among the 34 included patients, 19 (61.3%) underwent monotherapy for IBD; corticosteroid therapy was administered in 10 cases (32.3%), TNF antagonist therapy in 6 cases (19.4%), ustekinumab therapy in 2 cases (6.4%), and mesalazine therapy in 1 case (3.2%). A total of 11 patients (35.5%) received combination therapy, and one patient did not receive any treatment. Six patients failed in treatment with corticosteroids alone or in combination with mesalazine; they obtained significant improvement when treatment was changed to infliximab alone or in combination with corticosteroids (Table 5). All patients showed improvement, except for two patients who relapsed after 2 and 3 months of remission, respectively. Symptom improvement occurred between 2 days and 3 months after discontinuation of IL-17 inhibitor therapy, with three patients (20.0%) experiencing improvement within 2 weeks, seven patients (46.7%) between 2 and 4 weeks, and five patients (33.3%) showing improvement after more than 4 weeks. The median time to remission after discontinuation of IL-17 inhibitor treatment was 4 weeks. After discontinuation of IL-17 inhibitors, three patients (17.7%) switched to a TNF antagonist, five patients switched to ustekinumab (35.3%), and four patients (23.5%) selected another type of monoclonal antibody, such as tildrakizumab or guselkumab.

## Discussion

To the best of our knowledge, this is the first real-life comparative safety study using data from the FAERS database that assessed onset or exacerbation of IBD and colitis associated with all three IL-17 inhibitors in current use. Our study has provided supportive disproportionality analysis and data on the clinical characteristics of these rare adverse events. We found, from the perspective of pharmacovigilance, that the IL-17 inhibitors SEC (ROR = 2.13, 95% CI [1.96-2.30]) and IXE (ROR = 2.79, 95% CI [2.39-3.27]) can trigger IBD, while BRO did not trigger safety signals. We have also provided practical information about IBD and colitis types, time to symptom onset, and outcomes of treatment with IL-17 inhibitors, which had not been the focus of previous studies. A total of 388 case reports of primary suspect IL-17 inhibitor-related gastrointestinal inflammatory conditions were identified in the FAERS database, which included 268 IBD diagnoses and 120 colitis diagnoses. We detected four cases associated with BRO treatment, whereas the number of IBD cases that occurred after SEC treatment was 10 times greater than that observed after IXE therapy (89.7% vs. 9.3%). In a post-marketing study *via* VigiBase in 2021, no IBD was detected among patients in the BRO group, and the number of IBD cases associated with SEC treatment was five times that observed in the IXE treatment group (83.3% vs. 16.2%) (Petitpain et al., 2021), which is consistent with our findings. The number of IBD cases among patients treated with SEC, IXE, and BRO could, in part, be traced back to the different launch data (January 2015, April 2016, and July 2017, respectively). It is also worth noting that IL-17 inhibitors are not identical as they differ in pharmacology and target molecules. IXE is a humanized IgG4 antibody that has strong affinity for IL-17A, while SEC and BRO are fully humanized antibodies. Both SEC and IXE work by inhibiting IL-17A homodimers and IL-17A/F heterodimers, while BRO blocks the IL-17 receptor, thereby blocking all IL-17 isoforms and exerting a broader, less targeted effect (Fauny et al., 2020a). These data provide a potential explanation for the lower number cases of IBD associated with IXE or BRO therapy compared to treatment with SEC.

To date, studies based on pooled data from clinical studies, observational studies, retrospective studies, and meta-analyses have not provided substantial detail regarding the clinical features of IBD induced by IL-17 inhibitors (Onac et al., 2021; Schreiber et al., 2019; Caron et al., 2022). Our study has provided separate analyses of initial symptom onset, laboratory features, colonoscopy results, histopathological examination, treatment, and outcome. By collecting medical records from published case reports and case series involving IL-17 inhibitor-associated IBD, we revealed that diarrhea, bloody diarrhea, abdominal pain, and fever were the most common symptoms, which were often accompanied by elevated WBC count, ESR, C-reactive protein, and fecal calprotectin level. A systematic review concluded that gut histological and macroscopic mucosal inflammation occurred in up to 100% of patients with rheumatological disease, and 80% of these patients had increased fecal calprotectin levels (Fauny et al., 2020b), revealing that fecal calprotectin may act as a useful and non-invasive marker to predict IBD in patients with SpA or AS. Therefore, fecal calprotectin should be monitored to guide physicians’ decisions and enable stratification of patients to ensure optimal treatment. We also summarized colonoscopy and histopathological examination data from the included cases. According to the literature, ileocolonoscopy allows an accurate diagnosis of Crohn’s disease or ulcerative colitis in 90% of cases (Passos et al., 2018); therefore, it is recommended that patients with diarrhea, abdominal pain, bloody stools, or fever should be alerted to the possibility of IBD and undergo colonoscopy during IL-17 inhibitor treatment.

In the descriptive analysis, we observed that 91.2% of the cases were new-onset IBD, and about half of the new-onset cases were detected within 3 months of starting anti-IL-17 therapy. A study by Deodhar et al. (2019) reported that 30 of the 41 retrieved IBD cases (73.2%) were new-onset cases (Deodhar et al., 2019). Another analysis based on VigiBase data indicated that 91.8% of cases were new-onset IBD, and approximately three-quarters of IBD relapse cases were detected within 3 months of starting anti-IL-17 therapy (Petitpain et al., 2021). Given the long half-life of IL-17 inhibitors, the relatively short interval between drug use and IBD onset revealed an early impact of the drug on disease symptoms. However, it remains difficult to demonstrate whether anti-IL-17 drugs are coincidentally present or potential causative inducers of new or latent IBD. Previous research had demonstrated that patients with Pso, PsA, or AS may be at higher risk of developing IBD and that populations of patients with these rheumatologic diseases and populations with IBD have considerable overlap (Fieldhouse et al., 2020). In a genome-wide association study, several IBD loci were identified as overlapping with those of other immune-mediated inflammatory diseases (IMIDs), most notably AS and Pso (Jostins et al., 2012). The prevalence of IBD in Pso is estimated to be 1%–2% compared to 0.4% in the general population (Eppinga et al., 2017). Therefore, some Pso patients might have subclinical IBD triggered by IL-17 inhibitors. Since no recommendations are available regarding the management of patients with a history of IBD before starting an anti-IL-17 drug, it is of great importance to complete comprehensive patient history assessments, including the patient’s personal and family history of inflammatory bowel disease and other IMIDs before initiating treatment to help guide selection of the most appropriate management options. In recent years, the number of studies describing the correlation between slow and rapid onset of IBD, following IL-17 inhibitor treatment, has gradually increased. However, the pathogenesis of these paradoxical reactions after IL-17 administration remains unclear. The pathophysiology behind IBD involves upregulation of proinflammatory and immune-regulatory cytokines in the mucosa of the small and large intestines. IL-17 is a cytokine that is thought to contribute to the development of IBD and has strong proinflammatory activity (Magyari et al., 2014), which is significantly elevated in patients with active ulcerative colitis and Crohn’s disease (Seiderer et al., 2008). A previous study in mice has shown that blocking IL-17 leads to worsening of colitis by increasing tumor necrosis factor-α, interferon-γ, IL-6, and cytokines that promote the inflammatory response (Ogawa et al., 2004). Furthermore, IL-17A or IL-17RA inhibition has been associated with severe weakening of the intestinal epithelial barrier and reduction of intestinal epithelial integrity, which causes leakage of microorganisms and inflammation (Maxwell et al., 2015). However, it is important to note that IL-17 might also have beneficial effects within the intestinal tract. In animal models, gut-protective IL-17A was shown to be independently produced in the colonic lamina propria, where it promoted epithelial barrier function by regulating tight junction proteins after acute intestinal injury (Lee et al., 2015). This suggests a delicate balance between potentially favorable effects and detrimental effects orchestrated by IL-17 in the gut.

At present, there is no clinical guidance for the management of patients with disease flare or new-onset IBD after IL-17 inhibitor therapy. Our study suggests that alternative treatments to improve gastrointestinal symptoms and treatment of the underlying pathology have included mainly corticosteroids and TNF antagonists, as either monotherapy or combination therapy; mesalazine and other biological agents (such as ustekinumab) were shown to be effective as well. Corticosteroids were used in 66.7% of cases in our study, and the type, dose, and duration in each case depended on the improvement of symptoms. Discontinuation of IL-17 inhibitors resulted in substantial improvement of symptoms in all patients, except for two who experienced relapse after 2 or 3 months of remission. Generally, the prognosis of IBD related to IL-17 inhibitors was excellent, and we observed that most patients (66.7%) recovered within 4 weeks. However, we still recommend that these patients be followed up for a longer period to monitor for potential recurrence.

## Conclusion

Our retrospective analysis demonstrated that use of IL-17 inhibitors is associated with exacerbation or new-onset of IBD and colitis within 6 months of therapy. Rheumatologists and dermatologists should be on alert for gastrointestinal symptoms, such as diarrhea, bloody diarrhea, abdominal pain, and fever, along with elevated WBC count, ESR, C-reactive protein, and fecal calprotectin levels during IL-17 inhibitor therapy. It is essential to complete a comprehensive patient history prior to the initiation of treatment, include appropriate workup to monitor intestinal inflammatory markers, such as fecal calprotectin, and perform colonoscopy during treatment to help identify early symptoms and prevent severe complications. Early detection of adverse events *via* prompt evaluation, cessation of IL-17 inhibitors, and initiation of alternative treatment (e.g., corticosteroids and anti-TNF therapy) can generally lead to clinical remission of IBD. Further prospective studies are needed to evaluate and confirm risk factors for development of IBD associated with IL-17 inhibitor therapy.

## Data Availability

The raw data supporting the conclusions of this article will be made available by the authors, without undue reservation.

## References

[B1] Achufusi T. G. Harnee P. S. Rawlins S. (2019). A rare case of new-onset ulcerative colitis following initiation of secukinumab. Case Rep. Med. 2019, 2975631–2975635. 10.1155/2019/2975631 31467555 PMC6701396

[B2] Ali A. K. Torosian A. Porter C. Bloomfeld R. S. Feldman S. R. (2021). New onset inflammatory bowel disease in patient treated with secukinumab: Case report and review of literature. Dermatol Ther. 34, e15151. 10.1111/dth.15151 34609037

[B3] Burisch J. Eigner W. Schreiber S. Aletaha D. Weninger W. Trauner M. (2020). Risk for development of inflammatory bowel disease under inhibition of interleukin 17: A systematic review and meta-analysis. PLOS ONE 15, e0233781. 10.1371/journal.pone.0233781 32459816 PMC7252630

[B4] Caron B. Jouzeau J. Y. Miossec P. Petitpain N. Gillet P. Netter P. (2022). Gastroenterological safety of IL-17 inhibitors: A systematic literature review. Expert Opin. Drug Saf. 21, 223–239. 10.1080/14740338.2021.1960981 34304684

[B5] Darch K. M. Holland T. L. Spelman L. J. (2020). Secukinumab-induced inflammatory bowel disease in a patient treated for chronic plaque psoriasis and psoriatic arthritis: A case report and review of the role of novel biologic agents targeting the p19 subunit of IL-23. Case Rep. Med. 2020, 9404505. 10.1155/2020/9404505 32774388 PMC7395993

[B6] Deodhar A. Mease P. J. McInnes I. B. Reich K. BlAuvelt A. (2019). Long-term safety of secukinumab in patients with moderate-to-severe plaque psoriasis, psoriatic arthritis, and ankylosing spondylitis: Integrated pooled clinical trial and post-marketing surveillance data. Arthritis Res. Ther. 21, 111. 10.1186/s13075-019-1882-2 31046809 PMC6498580

[B7] Ehrlich D. Jamaluddin N. Pisegna J. Padua D. (2018). A challenging case of severe ulcerative colitis following the initiation of secukinumab for ankylosing spondylitis. Case Rep. Gastrointest. Med. 2018, 9679287–9679294. 10.1155/2018/9679287 29666723 PMC5832071

[B8] Eppinga H. Poortinga S. Thio H. B. Nijsten T. E. C. Nuij V. J. A. A. van der Woude C. J. (2017). Prevalence and phenotype of concurrent psoriasis and inflammatory bowel disease. Inflamm. Bowel Dis. 23, 1783–1789. 10.1097/MIB.0000000000001169 28617755

[B9] Fauny M. D'Amico F. Bonovas S. Netter P. Danese S. Loeuille D. (2020). Faecal calprotectin for the diagnosis of bowel inflammation in patients with rheumatological diseases: A systematic review. J. Crohns Colitis 14, 688–693. 10.1093/ecco-jcc/jjz205 31858121

[B10] Fauny M. Moulin D. D'Amico F. Netter P. Petitpain N. Arnone D. (2020). Paradoxical gastrointestinal effects of interleukin-17 blockers. Ann. Rheum. Dis. 79, 1132–1138. 10.1136/annrheumdis-2020-217927 32719044

[B11] Felice C. Leccese P. Scudeller L. Lubrano E. Cantini F. Castiglione F. (2019). Red flags for appropriate referral to the gastroenterologist and the rheumatologist of patients with inflammatory bowel disease and spondyloarthritis. Clin. Exp. Immunol. 196, 123–138. 10.1111/cei.13246 30554407 PMC6422654

[B12] Fieldhouse K. A. Ukaibe S. Crowley E. L. Khanna R. O'Toole A. Gooderham M. J. (2020). Inflammatory bowel disease in patients with psoriasis treated with interleukin-17 inhibitors. Drugs Context 9, 2020. 10.7573/dic.2020-2-1 PMC7185907 32362930

[B13] Fobelo L. M. Serrano G. R. Castro F. M. (2018). Emergence of inflammatory bowel disease during treatment with secukinumab. J. Crohns Colitis 12, 1131–1133. 10.1093/ecco-jcc/jjy063 29746636

[B14] Gossec L. Baraliakos X. Kerschbaumer A. de Wit M. Mclnnes I. Dougados M. (2020). EULAR recommendations for the management of psoriatic arthritis with pharmacological therapies: 2019 update. Ann. Rheum. Dis. 79, 700–712. 10.1136/annrheumdis-2020-217159 32434812 PMC7286048

[B15] Griffiths C. E. Reich K. Lebwohl M. van de Kerkhof P. Paul C. Menter A. (2015). Comparison of ixekizumab with etanercept or placebo in moderate-to-severe psoriasis (UNCOVER-2 and UNCOVER-3): Results from two phase 3 randomised trials. Lancet 386, 541–551. 10.1016/S0140-6736(15)60125-8 26072109

[B16] Grimaux X. Leducq S. Goupille P. Aubourg A. Miquelestorena-Standley E. Samimi M. (2018). Aphthous mouth ulcers as an initial manifestation of sécukinumab-induced inflammatory bowel disease. Ann. de Dermatologie de Vénéréologie. 145, 676–682. 10.1016/j.annder.2018.07.009 30366718

[B17] Grossberg L. B. (2019). A case report of successful treatment of Crohn's disease and psoriasis with guselkumab. Inflamm. Bowel Dis. 25, e84. 10.1093/ibd/izz033 30863855

[B18] Haidari W. Al-Naqshabandi S. Ahn C. S. Bloomfeld R. S. Feldman S. R. (2019). Asymptomatic Crohn’s disease identified in a patient being treated with secukinumab: A case report. SAGE Open Med. Case Rep. 7, 2050313X19893580. 10.1177/2050313X19893580 PMC6900620 31839950

[B19] Johnston D. N. Veettil R. (2019). A case of new onset ulcerative colitis following secukinumab treatment. Br. J. Hosp. Med. (Lond). 80, 544–545. 10.12968/hmed.2019.80.9.544 31498681

[B20] Jostins L. Ripke S. Weersma R. K. Duerr R. H. McGovern D. P. Hui K. Y. (2012). Host-microbe interactions have shaped the genetic architecture of inflammatory bowel disease. Nature 491, 119–124. 10.1038/nature11582 23128233 PMC3491803

[B21] Kukol W. Aranez L. Marino D. (2019). P055 development of Crohn’s disease with use of secukinumab. Am. J. Gastroenterol. 114, S15. 10.14309/01.ajg.0000578292.95094.87

[B22] Langley R. G. Elewski B. E. Lebwohl M. Reich K. Griffiths C. E. M. Papp K. (2014). Secukinumab in plaque psoriasis--results of two phase 3 trials. N. Engl. J. Med. 371, 326–338. 10.1056/NEJMoa1314258 25007392

[B23] Lebwohl M. Strober B. Menter A. Gordon K. Weglowska J. Puig L. (2015). Phase 3 studies comparing brodalumab with ustekinumab in psoriasis. N. Engl. J. Med. 373, 1318–1328. 10.1056/NEJMoa1503824 26422722

[B24] Lee A. Levell N. J. Shah S. N. Gaffney K. Tremelling M. (2020). Severe colitis complicating secukinumab (Cosentyx®) therapy. Clin. Exp. Dermatol 45, 344–345. 10.1111/ced.14149 31854471

[B25] Lee J. S. Tato C. M. Joyce-Shaikh B. Gulen M. F. Gulan F. Cayatte C. (2015). Interleukin-23-Independent IL-17 production regulates intestinal epithelial permeability. Immunity 43, 727–738. 10.1016/j.immuni.2015.09.003 26431948 PMC6044435

[B26] Ma J. Liu M. Liu Y. Wang N. (2022). Ulcerative colitis associatied with Secukinumab. J. adverse drug React. 24, 380–382.

[B27] Magyari L. Kovesdi E. Sarlos P. Javorhazy A. Sumegi K. Melegh B. (2014). Interleukin and interleukin receptor gene polymorphisms in inflammatory bowel diseases susceptibility. World J. Gastroenterol. 20, 3208–3222. 10.3748/wjg.v20.i12.3208 24695754 PMC3964393

[B28] Marin M. Alzueta N. Pío M. Gascón A. Castresana M. (2020). Ulcerative colitis induced by ixekizumab: A case report. Eur. J. Hosp. Pharm. 28, 50–52. 10.1136/ejhpharm-2019-002016 PMC7788206 33355284

[B29] Maxwell J. R. Zhang Y. Brown W. A. Smith C. L. Byrne F. R. Fiorino M. (2015). Differential roles for interleukin-23 and interleukin-17 in intestinal immunoregulation. Immunity 43, 739–750. 10.1016/j.immuni.2015.08.019 26431947

[B30] Menter A. Strober B. E. Kaplan D. H. Kivelevitch D. Prater E. F. Stoff B. (2019). Joint AAD-NPF guidelines of care for the management and treatment of psoriasis with biologics. J. Am. Acad. Dermatol 80, 1029–1072. 10.1016/j.jaad.2018.11.057 30772098

[B31] Merino Gallego E. Gómez Torres K. Martínez Amate E. (2020). Debut of inflammatory bowel disease associated to ixekizumab in patient with moderate, difficult -To-Manage psoriasis. Gastroenterol. Hepatol. 43, 622–623. 10.1016/j.gastrohep.2020.04.009 32674878

[B32] Mu X. Fardy J. Reid S. Trahey J. (2021). Severe drug-associated colitis with Crohn’s features in setting of ixekizumab therapy for chronic plaque psoriasis. Bmc Gastroenterol. 21, 361. 10.1186/s12876-021-01936-w 34600483 PMC8487480

[B33] Nallapeta N. Picano J. Bou-Abdallah J. (2019). Delayed onset of inflammatory bowel disease during treatment with secukinumab: 2112. Am. J. Gastroenterol. 114, S1176. 10.14309/01.ajg.0000597980.32774.79

[B34] Nazarian A. Grin A. Wijeratne D. T. (2020). Ixekizumab associated new-onset inflammatory bowel disease. ACG Case Rep. J. 7, e00316. 10.14309/crj.0000000000000316 32440523 PMC7209798

[B35] Obeidat A. E. Murakami T. T. (2021). New-onset collagenous colitis in a patient with psoriatic arthritis: Can it Be secukinumab? Cureus 13, e16147. 10.7759/cureus.16147 34367765 PMC8330501

[B36] Ogawa A. Andoh A. Araki Y. Bamba T. Fujiyama Y. (2004). Neutralization of interleukin-17 aggravates dextran sulfate sodium-induced colitis in mice. Clin. Immunol. 110, 55–62. 10.1016/j.clim.2003.09.013 14962796

[B37] Onac I. A. Clarke B. D. Tacu C. Lloyd M. Hajela V. Batty T. (2021). Secukinumab as a potential trigger of inflammatory bowel disease in ankylosing spondylitis or psoriatic arthritis patients. Rheumatol. Oxf. 60, 5233–5238. 10.1093/rheumatology/keab193 33677579

[B38] Ouyang W. Kolls J. K. Zheng Y. (2008). The biological functions of T helper 17 cell effector cytokines in inflammation. Immunity 28, 454–467. 10.1016/j.immuni.2008.03.004 18400188 PMC3424508

[B39] Passos M. Chaves F. C. Chaves-Junior N. (2018). The importance of colonoscopy in inflammatory bowel diseases. Arq. Bras. Cir. Dig. 31, e1374. 10.1590/0102-672020180001e1374 29972402 PMC6044200

[B40] Paul N. Hektner K. Bing Z. (2018). Secukinumab-induced unmasking of crohn colitis: A case report: 1509. Am. J. Gastroenterol. 113, S866–S867. 10.14309/00000434-201810001-01509

[B41] Petitpain N. D'Amico F. Yelehe-Okouma M. Jouzeau J. Y. Netter P. Peyrin-Biroulet L. (2021) IL-17 inhibitors and inflammatory bowel diseases: A postmarketing study in Vigibase. Clin. Pharmacol. Ther. 110: 159–168. 10.1002/cpt.2155 33411953

[B42] Philipose J. Ahmed M. Idiculla P. S. Mulrooney S. M. Gumaste V. V. (2019). Severe de novo Ulcerative Colitis following Ixekizumab Therapy. Case Rep. Gastroenterology 12, 617–621. 10.1159/000493922 PMC6244035 30483039

[B43] Ramiro S. Nikiphorou E. Sepriano A. Ortolan A. Webers C. Baraliakos X. (2023). ASAS-EULAR recommendations for the management of axial spondyloarthritis: 2022 update. Ann. Rheum. Dis. 82, 19–34. 10.1136/ard-2022-223296 36270658

[B44] Rodriguez M. R. Vazquez M. J. Pallares M. H. (2019). The onset of ulcerative colitis during treatment with secukinumab: Can anti-IL-17a be a trigger for inflammatory bowel disease? Rev. Esp. Enferm. Dig. 111, 720–721. 10.17235/reed.2019.5841/2018 31333036

[B45] Schreiber S. Colombel J. F. Feagan B. G. Reich K. Deodhar A. A. McInnes I. B. (2019). Incidence rates of inflammatory bowel disease in patients with psoriasis, psoriatic arthritis and ankylosing spondylitis treated with secukinumab: A retrospective analysis of pooled data from 21 clinical trials. Ann. Rheum. Dis. 78, 473–479. 10.1136/annrheumdis-2018-214273 30674475 PMC6530077

[B46] Seiderer J. Elben I. Diegelmann J. Glas J. Stallhofer J. Tillack C. (2008). Role of the novel Th17 cytokine IL-17F in inflammatory bowel disease (IBD): Upregulated colonic IL-17F expression in active Crohn's disease and analysis of the IL17F p.His161Arg polymorphism in IBD. Inflamm. Bowel Dis. 14, 437–445. 10.1002/ibd.20339 18088064

[B47] Sethi V. Jacobs A. Sethi A. (2019). P089 secukinumab induced ulcerative colitis in a patient with psoriatic arthritis: A novel approach to refractory cases. Am. J. Gastroenterol. 114, S23. 10.14309/01.ajg.0000613324.15639.1f

[B48] Shimizu K. Matsushita T. Takehara K. Hamaguchi Y. (2019). A case of juvenile localized scleroderma with anti-topoisomerase I antibody. Eur. J. Dermatol 29, 443–444. 10.1684/ejd.2018.3426 30442630

[B49] Shukla T. McCurdy J. Fahim S. Rostom A. (2018). A90 three patients with inflammatory bowel disease diagnosed while being treated with secukinumab for psoriasis. J. Can. Assoc. Gastroenterology 1, 135–136. 10.1093/jcag/gwy009.090

[B50] Smith M. K. Pai J. Panaccione R. Beck P. Ferraz J. G. Jijon H. (2019). Crohn’s-like disease in a patient exposed to anti-interleukin-17 blockade (ixekizumab) for the treatment of chronic plaque psoriasis: A case report. Bmc Gastroenterol. 19, 162. 10.1186/s12876-019-1067-0 31488067 PMC6727530

[B51] Sykaras A. G. Margellou E. Vallianou N. G. Panagopoulos F. Geladari E. Kounatidis D. (2022). Multifaceted secukinumab-induced colitis. Inflamm. Bowel Dis. 28, e47–e48. 10.1093/ibd/izab267 34792606

[B52] van der Heijde D. Ramiro S. Landewe R. Baraliakos X. Van den Bosch F. Sepriano A. (2017). 2016 update of the ASAS-EULAR management recommendations for axial spondyloarthritis. Ann. Rheum. Dis. 76, 978–991. 10.1136/annrheumdis-2016-210770 28087505

[B53] van Puijenbroek E. P. Bate A. Leufkens H. G. Lindquist M. Orre R. Egberts A. C. (2002). A comparison of measures of disproportionality for signal detection in spontaneous reporting systems for adverse drug reactions. Pharmacoepidemiol Drug Saf. 11, 3–10. 10.1002/pds.668 11998548

[B54] Vernero M. Astegiano M. Ribaldone D. G. (2019). New onset of inflammatory bowel disease in three patients undergoing IL-17a inhibitor secukinumab: A case series. Am. J. Gastroenterol. 114, 179–180. 10.1038/s41395-018-0422-z 30429591

[B55] Wang J. Bhatia A. Cleveland N. K. Gupta N. Dalal S. Rubin D. T. (2018). Rapid onset of inflammatory bowel disease after receiving secukinumab infusion. ACG Case Rep. J. 5, e56. 10.14309/crj.2018.56 30105273 PMC6072803

[B56] Wendling D. Hecquet S. Fogel O. Letarouilly J. G. Verhoeven F. Pham T. (2022). 2022 French Society for Rheumatology (SFR) recommendations on the everyday management of patients with spondyloarthritis, including psoriatic arthritis. Jt. Bone Spine. 89, 105344. 10.1016/j.jbspin.2022.105344 35038574

