# Supplementary material for: IL-17 inhibitor-associated inflammatory bowel disease: A study based on literature and database analysis
Source: Front Pharmacol. 2023 Mar 23;14:1124628. doi: 10.3389/fphar.2023.1124628 (PMC10076642; doi:10.3389/fphar.2023.1124628)
Supplement: Supplementary file 1 [file Table1.docx]

**Supplementary Table S1**. Calculation of reporting odds ratio (ROR).

|  | Adverse drug event of interest | All other adverse drug events of interest |
| --- | --- | --- |
| Drug of interest | a | b |
| All other drugs of interest | c | d |

The calculation formulas are shown below:

1. ROR=ad/b/c
2. 95%CI=e^ln(ROR)±1.96(1/a+1/b+1/c+1/d)^0.5^
